# Transgene-free genome editing in plants

**DOI:** 10.1016/j.abiote.2026.100057

**Published:** 2026-05-21

**Authors:** Yue-Hao Gao, Qi Lin, Yu-Jing Wang, Yu Cao, Xin-Xin Li, Dian-Chen Yue, Hou-Ling Wang

**Affiliations:** State Key Laboratory of Tree Genetics and Breeding, School of Biological Sciences and Technology, Beijing Forestry University, Beijing, 100083, China

**Keywords:** Transgene-free, Ribonucleoprotein complexes, Base editing, Prime editing, mRNA delivery systems

## Abstract

Genome-editing tools for the precise, efficient modification of DNA have led to groundbreaking advances in crop improvement and basic plant science research. However, conventional genome editing may result in the integration of unintended gene fragments into the host genome, along with off-target effects and the risk of genetic drift of resistance genes. By contrast, transgene-free genome editing represents a revolutionary breakthrough due to its ability to 1) achieve precise and stable genomic modifications while minimizing foreign DNA integration through DNA-free or transient delivery of CRISPR components; and 2) deliver ribonucleoprotein complexes (RNPs) or mRNA into the host. In this review, we discuss the core principles of transgene-free genome-editing technologies, including the direct delivery of RNPs, mRNA delivery systems, the precise substitution of single nucleotides using cytosine or adenine base editors (CBEs, ABEs), and the mechanisms behind multiple types of prime editing that use templates for reverse transcriptases. These techniques have driven the development of crops considered nongenetically modified, as they do not contain stably integrated foreign DNA. Finally, we discuss future prospects, including the development of transgene-free genome-editing tools that combine delivery systems with artificial intelligence–assisted optimization, with promising applications for agriculture.

## 1 Introduction

Genome editing enables the generation of targeted genetic changes, such as base substitutions and small insertions/deletions, at specific loci in the host genome. Genome editing in plants has facilitated basic research into gene function and trait improvement in crops [[1], [2], [3], [4]]. During conventional genome editing, double-strand breaks (DSBs) are induced in plant genomic DNA. The repair mechanisms of cells, including nonhomologous end joining (NHEJ) and homology-directed repair (HDR), are then co-opted for gene modification. However, the delivery of DNA vectors using conventional Agrobacterium (*Agrobacterium tumefaciens*)-mediated genome editing can lead to the stable integration of T-DNA from Agrobacterium, complicating efforts for “transgene-free” editing to avoid regulatory constraints. The emergence of the clustered regularly interspaced short palindromic repeats (CRISPR)/CRISPR-associated nuclease 9 (Cas9) system serves as a powerful, accessible platform that has revolutionized the field of genome editing [4,5]. For example, *Agrobacterium*-mediated transient expression of *Cas9* and CRISPR components coupled with rapid, high-throughput screening allows non-transgenic edited plants to be recovered without relying on sexual segregation of the edits away from the transgene [6].

Transgene-free genome editing is increasingly being used for crop improvement due to its ability to generate targeted genetic changes without the stable integration of foreign DNA [[7], [8], [9]]. The successful delivery of ribonucleoprotein complexes (RNPs) marked the first major step in transgene-free editing. By directly delivering preassembled Cas9–sgRNA ribonucleoprotein (RNP) complexes or Cas9/sgRNA-encoding mRNAs (rather than DNA expression cassettes), the editing components act transiently and are typically cleared after editing, thereby minimizing the risk of stable foreign-DNA integration into the host genome [[10], [11], [12], [13], [14], [15]]. In parallel, precision editors such as base editors and prime editors have expanded the range of possible scarless modifications beyond DSB-dependent repair, enabling targeted substitutions and broader sequence changes [16,17]. The increasing demand for transgene-free editing arises from practical challenges in various fields. In agricultural biotechnology, transgene-free edited crops can often circumvent the stringent regulations associated with transgenes, thereby accelerating the commercialization of these crops, as they lack foreign transgenic components [3]. In the fields of biomedicine and basic research, an editing system for immediate expression of foreign genes reduces the risk of off-target effects and persistent mutation of the genome, thereby mitigating ethical concerns and meeting safety standards. Substantial progress has been made in the development of transgene-free crops with enhanced stress resistance. However, technical bottlenecks remain, including low editing efficiency, the lack of stable delivery systems, and difficulty in precisely editing large genomic fragments [18].

**Fig. 1:**
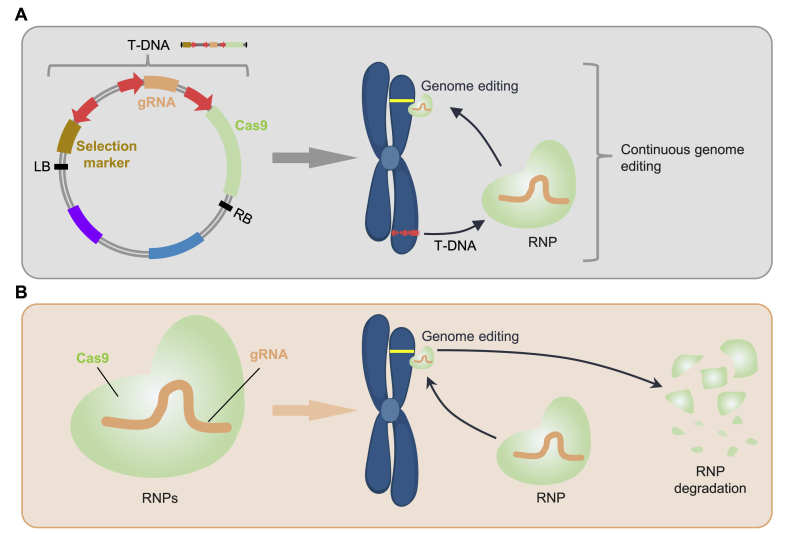
CRISPR and direct delivery of ribonucleoprotein (RNP) for genome editing. **A** Genome editing method using conventional CRISPR system. **B** A schematic diagram illustrating the direct delivery of RNP method for transgene-free genome editing.

Conventional editing via *Agrobacterium*-mediated transformation typically introduces CRISPR/Cas9 components as T-DNA expression cassettes, followed by selection and regeneration to obtain edited plants. Because the T-DNA largely inserts in the genome randomly, stable transformation can introduce unintended genomic disruptions and insertion-site ambiguity, which may complicate downstream analysis and breeding. By contrast, transgene-free genome-editing approaches aim to avoid stable integration of foreign DNA and to limit the duration of editor activity, which can reduce integration-associated genomic disruption and simplify downstream interpretation compared with DNA-vector–based stable transformation [19]. By directly delivering RNP complexes or mRNAs, transgene-free strategies can reduce integration-associated genomic interference and allow transient editor activity to be eliminated after editing (Fig. 1), which is particularly relevant for precision tools such as base editing and prime editing. Beyond DNA-free delivery, transient expression workflows may also be used, where editing cassettes are expressed temporarily without persistent genomic retention under controlled conditions, leading to the generation of edited plants with minimal residual foreign DNA. Accordingly, a central focus of transgene-free engineering is to balance editing activity with verifiable non-integration of foreign DNA rather than optimizing delivery parameters in isolation.

Several approaches can be taken to ensure that the final edited plants do not retain any foreign DNA sequences, including the use of DNA-free delivery systems, transient expression, or stable transformation followed by genetic segregation or programmed elimination of transgenic elements. In this review, we summarize advances in transient strategies and diverse delivery systems. We also provide a concise overview of key routes and representative selection methods used to achieve transgene-free editing through stable transformation. We summarize the technical principles, key breakthroughs, and practical applications of transgene-free genome editing in plants. Finally, we discuss the latest progress in transgene-free editing systems in plants, explore their potential applications in agriculture, and discuss future research directions in this exciting field.

## 2 Prime editing and single-base editing for transgene-free genome engineering

### 2.1 Prime editing

**Fig. 2:**
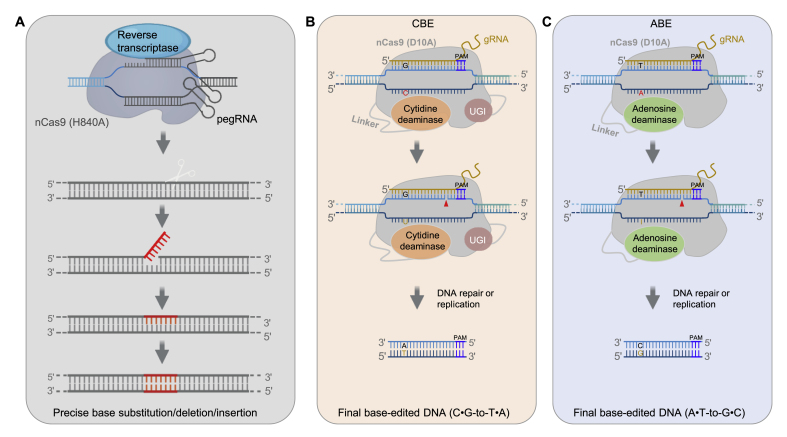
Prime editing (PE) and single base editing for transgene-free genome engineering. **A** A simple schematic representation of how prime editing works. The pegRNA contains the target sequence along with the RNA template that carries the editing information. Cas9 reverse transcriptase (RT) creates a single-stranded cut at the target DNA site and synthesizes a new DNA strand through reverse transcription, utilizing the pegRNA as a template. The cell employs the new strand to repair the original DNA and incorporate the desired sequence, allowing for the insertion, deletion, or replacement of multiple bases. The PBS depicted in the image refers to the primer binding site. **B–C** A schematic illustrating the mechanisms of the two single base editing systems: cytosine base editor (CBE) (**B**) and adenine base editor (ABE) (**C**). The C→T base editor (CBE) primarily consists of nCas9, cytidine deaminase, and uracil glycosylase inhibitor (UGI). The A →G base editor (ABE) primarily consists of nCas9 and adenine deaminase. Fusion proteins bind to target DNA, while deaminases convert specific bases (e.g., C to U or A to I) into intermediates. The DNA repair system recognizes uracil (U) as thymine (T) or inosine (I) as thymine (T) to facilitate base substitution, without the need for double-strand breaks.

Prime editing was developed to extend precise genome engineering beyond single-base substitutions achieved by conventional base editors and to enable a broader range of small sequence changes, including substitutions, insertions, and deletions [20,21]. The core prime-editing system consists of a fusion between a Cas9 nickase variant and a reverse transcriptase (nCas9–RT) and a prime-editing guide RNA (pegRNA) containing a spacer, a primer-binding site (PBS), and a reverse-transcription template. Prime editors (PEs) generate the desired edit without introducing a DSB [22,23] (Fig. 2). While this design is considered to be a flexible route to precision editing, its performance in plants strongly depends on component design, the proper delivery of the prime-editing system, and the ability to recover transgene-free edited plants.

PE1–PE3 are commonly used PE systems. PE1, which pairs nCas9–M-MLV RT with a pegRNA, has variable performance in different plant species and using different pegRNA designs (e.g., PBS/RT-template features) [24]. PE2 employs M-MLV RT variants to improve editing outcomes [24,25], whereas PE3 also adds a second nicking sgRNA to bias repair toward the edited strand, enhancing editing efficiency [17,26]. The PE3 system was used to edit the *Defective kernel 1* (*PpDEK1*) gene, which regulates gametophyte trophectoderm and bud development in the model moss *Physcomitrium patens*, achieving an editing efficiency as high as 1.5% and facilitating efficient mutant generation [27]. The PE system has also been successfully employed for efficient genome editing in rice (*Oryza sativa*) and wheat (*Triticum aestivum*) [19,21,28]. Overall, these studies support the feasibility of these techniques. However, the efficiency and reproducibility of PE-mediated genome editing can vary substantially across species and loci. The optimization of regulatory elements and construct design has been explored to improve editing outcomes in plants including dicots [29]. PE3 constructs carrying dual pegRNA vectors have been used to edit the green fluorescent protein (*GFP*) gene in legumes such as peanut (*Arachis hypogaea*), chickpea (*Cicer arietinum*), and cowpea (*Vigna unguiculata*), with overall editing efficiencies of 0.2–0.5% [30]. Prime editing has also been combined with selectable/proxy loci (e.g., *Acetolactate synthase* [*ALS*]-related alleles conferring herbicide resistance) to facilitate the enrichment and recovery of edited events, which is particularly useful for transgene-free workflows [[31], [32], [33], [34]]. Importantly, prime-edited transgene-free rice plants have also been generated in a single step via transient delivery [35]. Therefore, in addition to discussing different PE variants, Table 2 lists end-to-end workflows that link delivery, recovery, and evidence of non-integration.

### 2.2 Base editing

Base editing is used to create targeted base conversions at defined loci without the need for donor DNA and typically without inducing DSBs, making it a practical route for precise allele engineering in plants [23]. Common platforms include cytosine base editors (CBEs), adenine base editors (ABEs), and C-to-G base editors (CGBEs), which primarily differ in the deaminase/repair modules used to target specific bases for modification [[36], [37], [38]]. While these editors broaden the scope of precise editing of specific sequences, their suitability for transgene-free engineering hinges on how they are delivered, recovered, and their lack of integration into the host genome validated.

A key challenge for using DNA-free or transient systems is the lack of sustained selection pressure. Therefore, endogenous selectable loci have been leveraged to enrich edited events without the need to introduce antibiotic or fluorescent marker genes. *ALS* serves as a widely used proxy locus because ALS-inhibiting herbicides block branched-chain amino acid biosynthesis, and specific point mutations in *ALS* confer herbicide resistance [39,40]. CBEs have been employed to introduce herbicide-resistant alleles and facilitate the enrichment of edited regenerants, leading to the generation of transgene-free plants in early generations [41,42]. Similarly, CBE-mediated editing of *AhALS2* in peanut to generate the *AhALS2*^P197S^ allele led to the production of herbicide-resistant plants at an editing efficiency of 3.5% with no detectable off-target effects [43]. These examples illustrate how selectable/proxy loci can partially compensate for limited selection pressure in transient delivery systems.

Single-base editing has been widely utilized in various studies. The base editors CBE3 and CBE4 have been extensively employed in hybrid poplar, tomato (*Solanum lycopersicum*), potato (*Solanum tuberosum*), rice, wheat, and maize (*Zea mays*), achieving an editing efficiency as high as 64% in potato [15,[44], [45], [46]] (Table 1). Efficient base editing using SpCas9-based systems typically requires the presence of a canonical NGG protospacer adjacent motif (PAM) near the target site, decreasing their widespread use [37]. A new ABE was developed using the SpCas9-NG variant, which recognizes NG PAM sequences, thereby overcoming the strict dependence on canonical NGG-type PAMs. This advancement enabled A-to-G substitutions at the *OsSPL17* locus in rice, where NG, NAG, and NGCG PAM sequences were successfully recognized by the SpCas9-NG variant. The editing efficiency at *SQUAMOSA PROMOTER-BINDING PROTEIN-LIKE 17* (*OsSPL17*) reached 74.3%, significantly expanding the target range for base editing in rice [47,48]. Furthermore, the introduction of CGBEs has facilitated the diversification of editing sites. By optimizing the codon usage of three uracil-N-glycosylase enzymes derived from human UNG, *Escherichia coli* UNG, and uracil DNA glycosylase X (UDGX) from *Mycobacterium smegmatis*, the new CGBE vector achieved efficient C-to-G editing in rice, with efficiencies of up to 45.9% [49].

**Table 1:** Transgene-free genome editing in different species.

Species	Methods	Editor/Platform	Genes	Selection/Enrichment	References
Rice	Base editing	Base editor	*sgOs-siteX*	No explicit selection described	[48]
Rice, wheat	RNP	Nuclease (Cas RNP)	*OsDEP1*, *OsACC*, *TaALS*, *TaLOX2*	Herbicide-based selection/enrichment (ALS/AHAS)	[50]
*Dendrocalamus latiflorus, Phyllostachys edulis* Munro	*Bamboo mosaic* virus (BaMV)	Virus-mediated CRISPR delivery	*RDR6*	No explicit selection described	[51]
Soybean, tobacco	RNP	Nuclease (Cas RNP)	*FAD2*, *AOC*	No explicit selection described	[52]
Tobacco	Nanomaterials	Nanocarrier delivery	*mGFP5*, *ROQ1*	No explicit selection described	[53]
Tomato, soybean	Base editing	Base editor	*SlRIN*, SlMBP7, *GmHPPD*, *GmELF3A*, *GmFAD2*	No explicit selection described	[54]
Bread wheat	RNP	Nuclease (Cas RNP)	*TaGW2*, *TaGASR7*	No explicit selection described	[11]
Tobacco	Virus	Virus-mediated CRISPR delivery	*PDS*, *PCNA*	Phenotypic marker co-editing (PDS albino)	[55]
Wheat	RNP	Nuclease (Cas RNP)	*TaGASR7*, *TaDEP1*, *TaNAC2*, *TaPIN1*, *TaLOX2*	No explicit selection described	[10]
Rice	Base editing	Base editor	*OsALS1*	ALS/AHAS	[56]
Tobacco, tomato, pepper, crushed cherries, peanuts	Virus	Virus-mediated CRISPR delivery	*NbPDS*, *NbFucT*-1, *NbDCL2*	ALS/AHAS	[46]
Rice	RNP	Nuclease (Cas RNP)	*ALS*	ALS/AHAS	[57]
Maize	RNP	Nuclease (Cas RNP)	*LIG*, *ALS2*, *MS26*, *MS45*	ALS/AHAS	[58]
The grape (*Vitis vinifera* L.) variety is Nebbiolo	RNP	Nuclease (Cas RNP)	*PDS*	Phenotypic marker co-editing (PDS albino)	[59]
Tobacco	Virus	Virus-mediated CRISPR delivery	*GFP*, *PDS*, *RDR6*, *SGS3*	Phenotypic marker co-editing (PDS albino)	[12]
Wheat	Virus	Virus-mediated CRISPR delivery	*TaPDS*, *TaGW2*, *TaGASR7*	No explicit selection described	[60]
Wheat	RNP	Nuclease (Cas RNP)	*TaLOX2*	No explicit selection described	[61]
*Brassica napus* L.	RNP (PEG)	Nuclease (Cas RNP)	*BnaX.SGT.a*	No explicit selection described	[62]
Maize	Nanomaterials	Nanocarrier delivery	*gat*, *AmCyan1*, *DsRed2*	No explicit selection described	[63]
*Populus tremulaP. alba*	RNP	Nuclease (Cas RNP)	*CCR2*	No explicit selection described	[64]
*Oryza sativa*	Prime editing	Prime editor	*OsALS*, *OsEPSPS*, *OsCold1*, *OsCold1*	ALS/AHAS	[35]
Peanuts, chickpeas, cowpeas	Prime editing	Prime editor	*GFP*	No explicit selection described	[30]
Maize	Prime editing	Prime editor	*ZmALS1, ZmALS2*	ALS/AHAS	[31]
Tomato	Nanomaterials	Nanocarrier delivery	*GUS*	No explicit selection described	[65]
Hybrid poplar (*Populus tremula* × *P. alba*)	Base editing (*Agrobacterium* infection)	Base editor	*CCoAOMT1*, *ALS1*, *ALS2*	ALS/AHAS	[15]
*Arabidopsis thaliana*	Virus (TRV)	Virus-mediated CRISPR delivery	*AtPDS3*, *AtCHLII*	No explicit selection described	[66]

**Table 2:** A guideline for different transgene-free genome editing methods in plants by illustrating the advantages and disadvantages.

Methods		Advantages	Disadvantages	References
*Agrobacterium*-mediated	Prime editing	1. Wide range of species applications 2. Lots of useful target genes for editing (*ALS*) 3. High editing efficiency and accuracy 4. Variety of editing types 5. Technical complementarity with other editing tools	1. Limited editing efficiency 2. Difficult to design complex system 3. Not all sites can be edited efficiently 4. There are editing by-products, 5. Application of the technology is limited	[24] [26] [30] [23] [34] [20] [21] [27]
	Base editing	1. High precision 2. Simple to perform 3. Enables multiple editing 4. Facilitates wider sequence diversity 5. Wide range of applications	1. Limited scope due to PAM sequence restriction 2. May generate bystander mutations 3. Complex preparation of specific editor proteins 4. May trigger gene silencing 5. Editing efficiency is affected by many factors	[23] [54] [25] [67] [16] [44] [36] [62]
Protoplast- mediated	Liposome	1. Suitable for various goods (DNA, RNA, protein, RNP) 2. Relatively simple operation. 3. Efficient delivery method 4. The activity of the reagent can be protected. 5. little interference to cell physiology	1. Protoplast recovery problem 2. Diverse transfection efficiency. 3. The stability is influenced by many factors. 4. The cost is relatively high 5. Difficult to realize large-scale application.	[68] [40]
	Electroporation	1. Efficient reagent delivery capability 2. Suitable for a variety of goods types 3. Good repeatability. 4. Experimental conditions are easy to control. 5. Promote cell fusion	1. Protoplast preparation problem 2. Potential cell damage from high voltage pulses 3. Difficulty in plant regeneration 4. Higher equipment and technology requirements 5. Complex control of electrical pulse parameters	[69] [40]
	Cell penetrating peptide	1. Intracellular delivery can be realized 2. Suitable for plant cells with intact cell walls. 3. The mechanism is unique and diverse. 4. The delivery mode is relatively mild. 5. Can be combined with other technologies.	1. The specific mechanism has not been fully understood. 2. The application range is mainly concentrated in dicotyledonous plants. 3. Stability of cell penetrating peptide 4. The workload of screening and optimization is heavy. 5. Protoplast recovery problem	[70] [71] [72] [73] [74] [75]
	PEG	1. Higher transfection efficiency 2. Less damage to cells and protoplasts 3. Easy to operate (mixed culture) 4. Wide range of applications 5. Relatively low cost	1. Protoplast preparation problem 2. Difficulty in plant regeneration 3. May affect cell physiology 4. Transfection efficiency varies from plant to plant 5. The concentration of PEG has a significant influence	[40] [76] [77] [78]
RNP-related	Viral Vectors	1. Avoids the risk of transgene integration 2. Enables systemic infection and facilitates regulation and improvement of overall plant traits 3. Advantageous in carrying large fragments of gene editing elements and moving them within the host 4. Wide host range 5. Good biosecurity	1. Cargo capacity of vectors is small 2. Complexity of viral vector construction 3. Difficult to obtain non-chimeric plants 4. Antiviral agents affect regeneration efficiency 5. Dependency on tissue culture	[68] [46] [60] [79]
	Gene guns	1. Applicable to a wide range of species, varieties, and explants 2. Relatively easy to use, relies on force rather than pathogen 3. Leaves no trace of exogenous DNA 4. Enables rapid assessment 5. Reduces off-target mutations	1. May damage DNA, leading to chromosomal rearrangements 2. Deoxy ribonucleic acid may be unevenly loaded on particles 3. Genotype dependent 4. Higher equipment costs 5. Difficult to scale up production	[68] [40] [11] [57] [58]
	Nanomaterials	1. No need for brute force penetration of the cell wall 2. Can deliver multiple cargoes (DNA, RNA, RNP) 3. Wide variety of nanomaterials 4. Nanomaterials are highly modifiable 5. Potential for cell-specific delivery	1. Reagent delivery efficiency is limited 2. Lack of technological maturity 3. The effect on plant physiology is not clear. 4. Lack of standardized process 5. The contradiction between size and load	[80] [81] [82] [83]
	Epigenetic modification	1. Stabilizing gene expression regulation 2. Achieve methylation modification at specific gene loci 3. Multi-site targeting results in methylation of larger regions or multiple target genes to enhance gene silencing effects 4. Protect genomic stability 5. Multi-generational transmission of genetic information	1. Activation efficiency is affected by gRNA position, complicating experimental design 2. Transfection efficiency is limited 3. Unstable methylation level after transfection 4. Relative instability of epigenetic marks 5. Uncertainty due to reversibility	[84] [85] [86]

## 3 Protoplast-mediated regeneration for transgene-free genome editing

**Fig. 3:**
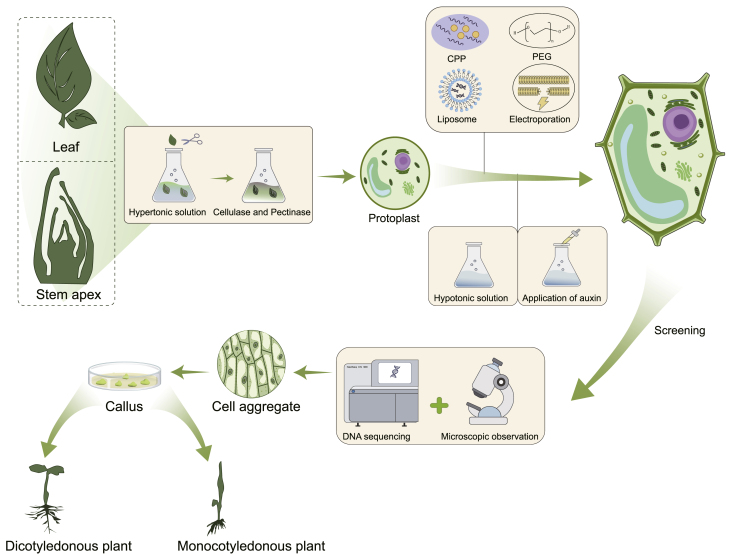
Protoplasts mediated regeneration for transgene-free genome editing. The schematic including the treatment of plant tissues to obtain protoplasts, the delivery of exogenous genes (plasmid or RNPs) using various methods (such as cell-penetrating peptides, the PEG method, liposomal delivery, and electroporation), the induced regeneration of the cell wall from the plant cell, and the subsequent tissue culture to produce a complete plant after microscopic observation and sequencing.

*Agrobacterium*-mediated genetic transformation can be performed using various methods, including the leaf disk method, vacuum infiltration, and co-culture [87,88]. Once the delivery of CRISPR components and genome editing are complete, obtaining edited plants becomes a critical issue. Genetically modified cells can be selected from young tissues or edited explants, and under aseptic conditions, the cell wall is digested with an enzymatic solution, and the mixture is filtered and centrifuged to collect protoplasts. The protoplasts begin to regenerate cell walls within approximately one week of culture and can form clusters of cells visible to the naked eye within approximately three weeks. After transferring the regenerated cell clusters to differentiation medium and selecting healthy callus, appropriate media are used to induce shoot and root regeneration to obtain complete plantlets through organogenesis or somatic embryogenesis, depending on the species and protocol. Following a period of acclimatization in open flasks, the plants can be transplanted and propagated (Fig. 3).

To overcome the complex, costly tissue culture procedure, tissue culture–free transformation methods have emerged. Common tissue culture–free techniques include microparticle bombardment, electroporation, the floral dip method, pollen tube passage, and infection of embryogenic tissue [88,89]. Genome editing through grafting [90] and the cut–dip–budding (CDB) method [87,91], in which *A. rhizogenes* is used to induce edited roots, provide tissue culture–free genome editing approaches; however, additional steps are required to ensure that regenerated shoots are free of integrated T-DNA sequences.

Advancements in tissue culture–free transformation methods will greatly simplify transformation, thereby reducing the number of transformation cycles and enhancing transformation efficiency, laying the foundation for molecular breeding and genetic research. Additionally, the removal of genome-editing components can be incorporated into the regeneration pipeline through inducible site-specific recombination systems, such as Cre/loxP or FLP/FRT, which enable precise excision of integrated editing cassettes after the desired mutation has been introduced. In addition, for methods that rely on stable expression vectors, the removal of foreign editing components can be integrated into the plant regeneration and selection procedure. For example, after targeted editing has been completed, inducible site-specific recombination systems (such as the Cre recombinase and its LoxP target sites) can be employed to excise the integrated CRISPR expression cassette from the genome [92]. This strategy allows the desired mutation to be retained while eliminating the editing construct, ultimately generating transgene-free plants, thus achieving the desired edit while eliminating any residual traces of foreign DNA. This method has been successfully employed for genome editing in various grape (*Vitis vinifera*) and apple (*Malus domestica*) varieties, including *VvMLO6* and *VvMLO7* in grape and *MdMLO7* in apple, achieving editing efficiencies greater than 90%. This approach provides an innovative strategy for addressing crop demands while adhering to regulations on transgenic materials [93,94].

## 4 Multiple delivery systems for transgene-free genome editing

### 4.1 mRNA and RNP delivery systems

mRNA delivery is a relatively straightforward, effective method for transgene-free genome editing. This approach involves delivering mRNAs encoding genome-editing tools (e.g., Cas9 or base editors) into host cells instead of using plasmids. The mRNA, synthesized through *in vitro* transcription (IVT), enters the cell directly via lipid nanoparticles (LNPs), particle bombardment, or other methods and is translated directly into proteins by ribosomes [68]. To date, mRNA delivery has been employed to edit *ALS*, *TaGASR7*, and other genes in wheat, achieving a maximum editing efficiency of 26.2% [10,50]. These mRNAs and their derived proteins are degraded in a timely manner to prevent sustained effects on the target site [95,96]. However, *in vitro*–transcribed Cas9 mRNAs can exhibit variable stability and translation efficiency *in vivo* due to differences in transcript length and secondary structure, leading to inconsistent editing outcomes when co-delivered with guide RNAs [95,97].

v2_TMV/DEN2, a recently developed mRNA delivery system, represents a new approach for improving the efficiency of mRNA delivery. By optimizing the 5′ untranslated region (5′ UTR) and the poly(A) tail of *in vitro*–transcribed mRNAs and by employing fisetin wrapping during particle bombardment, A-to-G and C-to-T base editing was achieved in rice suspension cells and immature wheat embryos. In this context, fisetin wrapping enhances mRNA stability during particle bombardment by protecting transcripts from degradation and promoting cellular uptake, thereby improving translation efficiency and overall editing outcomes. The efficiency of this system rose by 4.7-fold compared to the conventional plasmid delivery system [50]. In addition, intraplant particle bombardment (iPB) is a new wheat transformation technique that reduces the time required for genome editing by directly bombarding meristematic tissues in the embryonic stem tips of seeds. This method eliminates the need for antibiotic-mediated selection of callus cells and plant regeneration [61]. Although mRNA delivery has been successfully performed in several monocot plants, its use in other plant species remains challenging.

RNP delivery helps overcome the universal challenge of scarless editing in different plants. The RNP delivery system does not require transcription or translation. Cas9 and single-stranded sgRNA are preassembled into RNP complexes, which are then introduced into plant cells through physical or chemical methods. Following genome editing, the RNPs are rapidly degraded, allowing for target site modification while minimizing the likelihood of off-target effects [[98], [99], [100], [101]]. Indeed, Cas9–sgRNA RNPs demonstrated the same high editing efficiencies as plasmid-based expression systems [11,102]. Three methods are commonly used to deliver RNPs into cells: protoplast delivery systems, microparticle bombardment, and viral vectors.

### 4.2 RNP delivery into protoplasts

Transient expression in cells using RNPs is a widely employed technique in biology. However, unlike animal cells, plant cells possess a natural physical barrier, the cell wall, requiring a distinct approach for introducing biomolecules into these cells. Major components that facilitate the delivery of genome-editing agents in protoplasts include polyethylene glycol (PEG), cell-penetrating peptides (CPPs), liposomes, and electroporation.

The most common delivery strategy used in studies on protoplasts is mediated by PEG. This method involves the enzymatic removal of the cell wall to obtain protoplasts, followed by the introduction of CRISPR components via PEG-mediated transfection, leading to targeted editing in the protoplasts. In 2015, genome editing was demonstrated for the first time in rice, Arabidopsis (*Arabidopsis thaliana*), tobacco (*Nicotiana tabacum*), and lettuce (*Lactuca sativa*) protoplasts. Lettuce *bin2* mutants were regenerated at a rate as high as 46%, and no off-target mutations were detected in any of the mutants; *BRASSINOSTEROID-INSENSITIVE 2* (*BIN2*) encodes a negative regulator in the brassinosteroid signaling pathway [7]. PEG-mediated delivery of RNP complexes has also been demonstrated in grape and apple [103], *Petunia* × *hybrida* [104], and potato [105]. Transgenic plants can be generated from lettuce protoplasts, achieving a remarkable editing efficiency of 99%. This high efficiency allows precise site-directed mutations of chloroplast genes in lettuce, greatly facilitating in-depth studies of gene function. Additionally, the nuclease Cpf1 (also named Cas12a) has been incorporated into the plant RNP editing toolbox. LbCpf1/CRISPR RNA (crRNA) and AsCpf1/crRNA RNPs were successfully introduced into soybean (*Glycine max*) protoplasts, targeting two homologous genes, *FATTY ACID DESATURASE 2-1A* (*FAD2-1A*) and *FAD2-1B*, to increase oleic acid levels in soybean oil [58]. Fertilized eggs have been employed to improve plant traits through the use of PEG [76]. The authors stimulated the separation of rice sperm and egg cells *in vitro* to produce zygotes devoid of cell walls. CRISPR/Cas RNPs were then introduced into the cells and used to produce genome-edited zygotes. The edited zygotes developed into plants with stable inheritance of the edits.

Liposome-mediated transfection of protoplasts with CRISPR/Cas elements is similar to PEG-mediated transfection. However, unlike the PEG method, the liposome approach utilizes the properties of the plant cell membrane to deliver foreign substances relatively gently. Liposomes encapsulate RNPs through electrostatic binding and facilitate their entry into cells via endocytosis, aligning more closely with the characteristics of the cell membrane. The liposomes automatically dissociate after being delivered into the cell, supporting RNP activity. The use of lipid nanoparticles alone as a transfection reagent was recently demonstrated in grape. This approach circumvents the negative effect of liposomes combined with PEG on cell viability, showing promise for editing susceptibility genes to reduce pesticide usage in this crop [59]. The efficiency of various types of liposomes in delivering genome-editing agents differs. Lipofectamine 3000 and RNAiMAX achieved delivery efficiencies of 66% and 48%, respectively. Targeting the protoplasts of transgenic tobacco producing a fluorescent protein with these systems resulted in targeted mutagenesis at a rate of 6%, expanding the potential applications of liposome transfection [106].

CPPs are highly effective delivery systems for biomolecules. CPPs originally referred to as protein transduction domains (PTDs), are rich in basic amino acids such as arginine and lysine and typically consist of fewer than 30 amino acid residues. Like liposomes, CPPs are delivered into plant cells by direct penetration or endocytosis, thereby transporting biomolecules across plant cell walls and cell membranes into the protoplasts [72]. Direct penetration by CPPs (such as HIV-derived TAT peptide) creates transient pores by disrupting the arrangement of lipids, whereas endocytosis-mediated penetration of CPPs (such as Penetratin) involves translocation through endosomal escape via clathrin protein–mediated endocytosis. Ultimately, CPPs are degraded by endogenous cellular proteases [[73], [74], [75]]. CPPs can bind to proteins to achieve scarless editing. For example, a zinc finger nuclease (ZFN)–CPP complex was successfully used to deliver genome-editing components into wheat cells, resulting in effective editing of the *Inositol pentakisphosphate 2-kinase 1* (*IPK1*) gene. This approach is expected to reduce the phytic acid content and increase the nutritional value of feed grains [107]. The efficiency of CPP delivery varies among plant species. Among the CPPs tested, cationic dR9 (comprising two nine-arginine peptides) was the most efficient transporter in *Arabidopsis*, soybean, and tomato leaves sprayed with TAMRA (5-carboxytetramethylrhodamine) dye labeled with various CPPs [73]. Lipid nanoparticle (LNP) formulations were recently optimized by screening CPPs and various ionizable cationic lipids, further enhancing the efficiency of ABE and PE delivery [70].

Electroporation is a highly efficient delivery technique that uses a short-duration, high-voltage pulsed electric field (ranging from 100 to 1000 V/cm for several milliseconds) to polarize the lipid bilayer of the cell membrane, resulting in the formation of nanoscale hydrophilic pores. This process consists of three stages: 1) capacitive charging occurs, causing the electric field to drive ion migration and the potential difference across the membrane to exceed a critical threshold (approximately 1 V); 2) the phospholipid molecules rearrange to create a transient pore, facilitating delivery; 3) the electric field is removed, allowing the pore to self-repair due to membrane fluidity. Several parameters must be optimized to ensure successful electroporation. The introduction of Cre recombinase into tobacco cells via electroporation at a field strength of 50 V/cm led to the successful editing of a large fragment containing *GFP* flanked by loxP sequences, confirming the hypothesis that the recombination of target DNA sequences can be induced by electroporation under optimal conditions [69]. The importance of selecting the proper electroporation system and plant tissue should not be overlooked; for instance, the Neon electroporation system was used to edit the *CONSTITUTIVE PATHOGEN RESPONSE 5* (*CPR5*) gene in soybean protoplasts, achieving a maximum efficiency of 8.1%. Protoplasts derived from ternary leaves were shown to be the most effective for electroporation-mediated transformation [108].

### 4.3 Particle bombardment

**Fig. 4:**
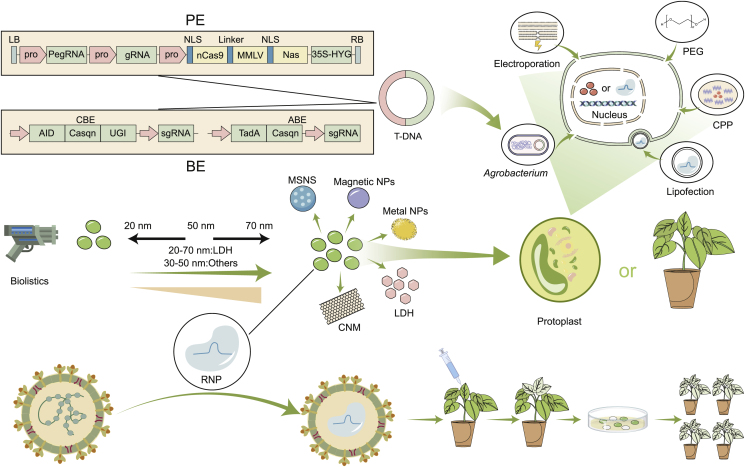
Transgene-free genome editing via multiple delivery systems. Transferring the plasmid containing T-DNA via *Agrobacterium*, which may include prime editing (PE) or base editing (BE). Transformation can also be accomplished by bombarding various types of particles, such as metal particles and nanomaterials, that carry exogenous DNA into plant tissues or protoplasts using a gene gun. Additionally, the artificial injection of viral vectors containing ribonucleoprotein (RNP) into plants can be performed, followed by screening in tissue culture to obtain fully transformed plants.

Particle bombardment is the preferred method for transferring genes in most crops. This method typically employs calcium chloride (CaCl_2_) and polyamine compounds, such as protamine, to coat micron- or even nanometer-sized gold or tungsten particles (Fig. 4). High-pressure gas (usually helium or nitrogen) provided by a gene gun accelerates these metal particles for penetration through plant cell walls and plasma membranes, delivering macromolecules such as proteins and DNA directly into the cytoplasm or nuclear region. Foreign DNA entering the nucleus can be integrated into the genome through NHEJ or homologous recombination (HR), resulting in stable genetic transformation. Even if the DNA is not integrated, efficient transient gene expression can still occur [[57], [63], [109]]. The target tissues commonly used in this technique include callus tissue, immature embryos, and other materials with strong regenerative capacity and active cell division [50]. Because particle bombardment does not rely on plant sensitivity to *Agrobacterium*, it is broadly applicable to poplar and other important tree species [10].

**Fig. 5:**
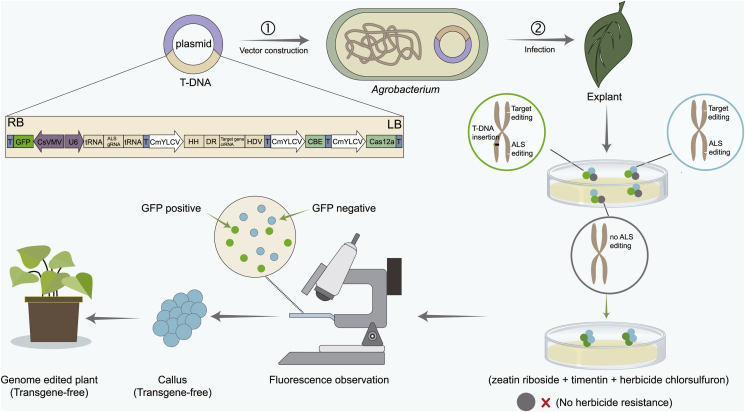
Transgene-free genome editing via using the *ALS* gene as an example by a double screening process. The CRISPR plasmid, which contains both the *GFP* and *ALS* genes, was transfected into *Agrobacterium* to infect the explants. The grey color points indicates cells that were not edited, while the blue and green cells both developed herbicide resistance. However, the blue cells underwent untraceable editing to activate the *ALS* gene, whereas the green cells incorporated the exogenous *GFP* gene, leaving behind detectable traces. Following herbicide screening, only the positive cell clusters remained. Subsequently, cells lacking green fluorescence were selected for tissue culture through fluorescence observation, ultimately yielding complete plants with *ALS* resistance achieved through transgene-free editing.

Particle bombardment–mediated delivery of RNP complexes into plant cells required the *in vitro* assembly of Cas-containing RNP complexes. These complexes are then coated with gold powder for delivery into plant cells. This technique was used to edit *ALS2* and male fertility genes *Male sterility 26* (*MS26*) and *MS45* in maize, resulting in an editing frequency of 2.4% to 9.7% in regenerated maize plants [58] (Fig. 5). The grain weight gene *TaGW2* and the grain length gene *TaGASR7* were successfully edited in immature wheat embryos via particle bombardment using RNP complexes targeting these two genes, resulting in transgene-free edited plants [11]. Additionally, a CRISPR/Cas transient expression vector targeting *TaGASR7* and *TaDEP1* was successfully introduced into wheat callus by particle bombardment, leading to the generation of gene-edited plants with a dwarf phenotype following differentiation and plant regeneration [10].

Nanotechnology has emerged as a powerful tool for traceless genome editing due to its highly adjustable physical and chemical properties, as well as excellent biocompatibility [83,53]. The primary advantages of nanomaterials include their controllable size (typically less than 100 nm), large specific surface area, and ease of surface functionalization (see below). These characteristics enable the efficient loading of CRISPR/Cas components (Cas9, sgRNA, and donor templates) and their controlled release in specific cells or tissues. Common types of nanomaterials include metal nanoparticles (such as gold and silver) [53,110], biodegradable polymers (such as polylactic-co-glycolic acid [PLGA]) [111], LNPs, carbon nanomaterials (including carbon dots and carbon nanotubes) [112], and DNA origami (self-assembled nanoscale-level nucleic acid structures) [113]. Importantly, small nanoparticles (<100 nm) have excellent cell-penetrating ability, eliminating the strong physical impact and risk of membrane rupture associated with traditional particles, and are suitable for plant materials with fragile structures such as protoplasts and callus [65]. Layered double hydroxide (LDH) nanoparticles 50 nm in diameter were used to deliver double-stranded RNA (*GUS*-dsRNA) into developing tomato pollen cells without the need for physical assistance or chemical treatment. At 3 days after treatment, *GUS* transcript levels had dropped by 89%, confirming successful delivery of the editing system [65].

Gold nanoparticles, known for their low toxicity, are frequently used in microparticle bombardment as alternatives to traditional micron-sized gold particles. This approach significantly reduces cytotoxicity and the risk of off-target effects while maintaining effective penetration [110,65]. The pCCR2-CRISPR plasmid was introduced into poplar tissues using DNA-encapsulated gold particles to edit *CINNAMOYL-CoA REDUCTASE 2* (*CCR2*), achieving an editing efficiency of up to 34.3%. Several transgene-free gene-edited plants were obtained [64]. Biodegradable polymers, such as PLGA, can slow the release of genome-editing elements and extend the expression time window, thereby improving editing efficiency [63,111]. By contrast, DNA origami allows the CRISPR/Cas9 system to precisely release editors at the target site in response to specific biological signals (e.g., microRNA abundance levels) through predesigned spatial configurations. This approach could enhance editing efficiency by achieving excellent cell selectivity and spatiotemporal controllability [114]. Additionally, carbon nanomaterials are widely used for transient expression in protoplasts and tissues due to their superior cell-penetrating ability and nonintegrative expression characteristics, making them suitable for scarless editing [53]. Single-walled carbon nanotubes (SWNTs) have also been employed as nanocarriers as they can noncovalently adsorb small-interfering RNA molecules on their surfaces, achieving silencing efficiency of *GFP* of up to 95% in tobacco [53].

The functionalized modification of nanomaterials is an important approach for gene delivery. By coupling specific peptide chains, antibodies, or glycan ligands to the surfaces of nanoparticles, the recognition and binding efficiency to particular cell types can be significantly enhanced. Moreover, the design of encapsulated pH-responsive or enzyme-responsive coatings allows the rapid release of nanoparticles in specific intracellular environments, thereby minimizing nontarget cell editing and off-target effects. For instance, microRNA-responsive nanocapsules released Cas9 exclusively in miR-21-accumulating cell, achieving a synergistic increase in targeting and editing accuracy [63,110,115]. Light- and magnetic-responsive nanomaterials might also be suitable for gene delivery. Using external stimuli such as near-infrared light and magnetic fields, the timing and location of gene release can be precisely regulated, which is especially suitable for studies of developmental regulation in plants that require tissue-specific or stage-specific expression [116]. The use of nanotechnology holds great potential for gene delivery and transgene-free editing in plants.

### 4.4 Viral transfection

Plant viruses with RNA genomes are also suitable for achieving transgene-free editing in plants due to the transient expression of viral particles and the nonintegrated nature of their genomes [77]. A tobacco rattle virus (TRV)-based system delivering a compact RNA-guided genome editor was successfully used for one-step, transgene-free germline editing in *Arabidopsis*, generating heritable edits, underscoring the potential of virus-mediated delivery beyond somatic tissues [66]. Single-stranded RNA viruses can be classified into positive-stranded viruses and negative-stranded viruses. Positive-stranded viruses rely on self-encoded RNA polymerase (RdRP) for replication and their transcripts are directly translated by host ribosomes to produce viral proteins. Due to their limited carrying capacity and instability during inheritance, insertions or deletions of fragments often occur when long nucleic acid sequences are delivered [117,118]. Consequently, positive-strand viruses are typically used to introduce some components of the CRISPR/Cas system (usually the sgRNA) into transgenic recipients that express *Cas*, thereby enabling the editing of plant genomes [12,46,117]. This approach has been validated in wheat and tobacco using delivery vectors for barley streak mosaic virus (BSMV) and TRV, which facilitated the direct delivery of sgRNA into germ cells or meristematic tissue cells of both species without the need for tissue culture, resulting in heritable genome editing in seeds derived from Cas-expressing transgenic plants, from which Cas9-free edited progeny can subsequently be obtained through genetic segregation [[55], [60], [79], [119]]. More recently, RNA virus–based delivery achieved transgene-free and tissue culture–free heritable genome editing in wheat, providing a proof-of-concept for monocot crops in which regeneration remains a major constraint [120]. Additionally, VITF-Edit technology was successfully employed to cross BSMV-sg-infected *Cas9*-expressing transgenic wheat pollen with wild-type wheat, leading to the generation of a *Cas9* transgene–free mutant [60].

Negative-strand RNA viruses exhibit strong genome stability and a significant cargo-carrying capacity because their linear nucleocapsid remains encapsulated during replication, preventing genome recombination [12,46,117,121,122]. In recently engineered systems, certain negative-strand RNA viruses have been shown to accommodate a complete CRISPR/Cas module, thereby eliminating the requirement for transgenic plants expressing *Cas9* and enabling transient genome editing without stable integration of foreign DNA into the host genome. Negative-strand RNA viruses are widely used for transgene-free editing in various plant species. The construction of the tomato spotted wilt virus (TSWV) vector represents a significant breakthrough. Following *Agrobacterium*-mediated infiltration of a construct based on TSWV, the genomes of tomato, sweet pepper (*Capsicum annuum*), and other crops were edited, achieving an impressive editing efficiency of 78.2% in tomato [46]. Furthermore, the insertion of the CRISPR/Cas module between the *N* and *P* genes of the negative-strand RNA virus chicory yellow-netted rhabdovirus (SYNV) led to the editing of *Phytoene synthase* (*PDS*, encoding a key enzyme in carotenoid biosynthesis) in tobacco, leading to an albino phenotype, with a mutation frequency for single-target editing reaching 91% [12]. Finally, editing using viral vectors combined with base editors has attracted increasing attention. Recombinant TSWV vectors have been employed to deliver CBEs and ABEs for editing in pepper, peanut, and other crops. CBE and ABE achieved conversion efficiencies of 29.4–60.8% and 12.8–25.2%, respectively, successfully combining single-base editing with viral vectors [123].

## 5 Selection methods

The presence or absence of sustained selection pressure represents a key factor for evaluating the suitability of genome-editing delivery strategies for generating transgene-free plants. Three types of CRISPR reagents are commonly used for plant genome editing: DNA-based CRISPR/Cas9 vectors, RNA molecules, and RNP complexes [124]. DNA vector–mediated delivery systems possess a distinct advantage in terms of selection, as selectable marker genes conferring antibiotic resistance or encoding fluorescent reporters can be integrated into the vector backbone. The use of the appropriate selective agents during tissue culture enriches cells carrying editing components, thereby greatly enhancing the efficient recovery of edited plants [10].

The isolation of genome-edited and transgene-free plants after stable transformation generally requires the delivery of CRISPR editing constructs together with selectable marker genes into recipient plant genomes, most commonly through *Agrobacterium*-mediated transformation [125], and the subsequent recovery of transgene-free edited plants through genetic segregation following sexual reproduction [2,126]. However, conventional identification methods require PCR amplification and genotyping, which are labor-intensive and time-consuming [127].

Multiple strategies have been developed to improve the efficiency of identifying transgene-free individuals following stable transformation. These approaches can be broadly divided into passive enrichment strategies and post-editing active elimination systems. Passive enrichment strategies include visual signal–assisted selection and growth-based selection. Fluorescent marker systems typically integrate genes encoding fluorescent proteins such as DsRed or GFP into CRISPR vectors, allowing the rapid identification of candidate transgene-free individuals using seedlings, or calli at the T0 stage or their derived seeds at the T1 stage [128]. Nevertheless, these systems require specialized fluorescence detection equipment, and fluorescence signals often show limited penetration through seed coats [129]. Pigment-based selection has also been explored. The RUBY system uses tyrosine, a metabolite widely present in plants, as a substrate that is converted to the red pigment betalain, making it suitable for many plant species [130,131]. However, the need to assemble multiple genes increases the complexity of vector construction. Moreover, pigment accumulation can be influenced by environmental conditions or developmental stage [132].

Growth-based selection relies on differential resistance or sensitivity to selection agents. Treatments generate phenotypic differences between plants carrying the editing construct and those lacking the construct following segregation, typically involving different survival rates or physiological responses. The effectiveness of this approach often depends on the genetic background of the plant. In addition, this approach might be stressful to the plant [133]. Examples include the CRISPR S system in rice, which combines an RNA interference cassette targeting the cytochrome P450 gene *CYP81A6*, a gene that confers tolerance to the herbicide bentazon; plants retaining the transgenic construct remain bentazon-tolerant, whereas segregants lacking the construct become sensitive, thereby enabling negative selection against transgenic individuals; the use of *PARAQUAT RESISTANT 1* (*PAR1*) as a selectable marker in *Arabidopsis*, with paraquat treatment used for screening in a single generation; and a rice system that employs transient hygromycin treatment followed by 3,3′-diaminobenzidine (DAB) staining to distinguish individuals based on differences in hydrogen peroxide accumulation [[67], [133], [134]].

Although passive enrichment strategies can shorten the time required to obtain transgene-free individuals, their theoretical segregation frequencies remain limited. Under the ideal scenario of a single insertion in a diploid genome, the expected proportion of transgene-free progeny is approximately 25% [135]. To overcome this limitation, active post-editing elimination systems have been developed.

The Transgene Killer CRISPR (TKC) system employs a *BARNASE* cytotoxic module driven by promoters such as the *RICE EMBRYO GLOBULIN 2* (*REG2*) promoter to selectively eliminate transgene-carrying gametes or embryos, thereby reducing the likelihood of their transmission [135]. However, low-frequency escape events have been reported [136]. An improved version, TKC2, incorporates a RUBY reporter module to enable visual identification of transgene-containing tissues, together with a multi-stage auto-elimination module designed to selectively remove transgene-positive gametes or embryos. Using this system, the recovery efficiency of transgene-free progeny approached 100% [137]. Nevertheless, the strong lethal effect of this system may restrict continuous editing across generations and increase the risk of partial editing or chimerism [135,136].

The Fluorescent Marker and Pollen Killer CRISPR (FMPKC) system combines the fluorescent protein DsRed2 with a pollen-specific cytotoxic module, typically driven by a gametophyte-specific promoter to express a lethal gene such as barnase, thereby selectively eliminating transgene-carrying pollen. This design enables visual segregation while retaining a subset of seeds carrying editing components, increasing the probability of obtaining plants that are fully edited and ultimately transgene-free [138]. However, the broader applicability of this system might be limited by variations in gamete-specific promoter activity across species. Moreover, this system remains challenging for woody plants with long breeding cycles.

RNA-based and RNP-based delivery systems rely on transient gene expression and do not involve genomic integration. These systems eliminate the need for the stable maintenance of selectable marker gene expression, ultimately leading to a general lack of effective selection pressure (Table 1). To address this limitation, two major screening strategies have been developed for these editing systems: molecular detection–based screening and PDS-based co-editing strategies [11,14,104]. Molecular detection approaches do not depend on marker genes. Instead, editing events are identified directly using molecular techniques, including restriction enzyme digestion, targeted deep sequencing, and high-resolution melting (HRM) analysis. However, these approaches require nucleic acid extraction, PCR amplification, and sequence verification for each regenerated plant or cell clone. This labor-intensive, time-consuming procedure is poorly suited for the large-scale screening of mutant populations [139].

PDS-based co-editing provides an effective alternative screening strategy. PDS encodes a key enzyme in the carotenoid biosynthesis pathway and is highly conserved across plant species; loss-of-function mutations typically result in an albino phenotype, offering a visual indicator of editing events [11]. In practice, editing reagents (RNA or RNP) targeting PDS can be co-delivered with those targeting the gene of interest, enabling rapid identification of double-positive edited plants through two steps comprising phenotypic pre-screening and molecular validation. Subsequent genetic segregation via selfing or backcrossing allows the wild-type *PDS* allele to be restored, ultimately yielding plants carrying the desired edits only in the target gene. Because PDS is an endogenous plant gene, this screening strategy avoids the introduction of foreign marker genes, thereby reducing regulatory concerns and public apprehension associated with transgenic approaches and aligning with the practical objectives of transgene-free genome editing.

## 6 Conclusions and perspectives

Transgene-free editing can be viewed as an evidence-based, workflow-level outcome. The central message of this review is that successful transgene-free editing is determined less by any single editor than by whether an end-to-end pipeline can repeatedly lead to molecularly clean, heritable genotypes in crops despite regeneration constraints. Across the major routes to transgene-free editing summarized in Table 1, a consistent trade-off emerges: delivery systems that are operationally controllable and efficient can increase concerns about molecular footprints and ambiguous outcomes, whereas more transient, footprint-minimizing strategies tend to shift the burden downstream into limitations in plant regeneration, variable outcomes across genotypes, and heavier screening demands that directly challenge scalability. Emerging delivery techniques broaden what is technically possible, but their value will ultimately be judged by their ability for standardization, predictability, and rigorous validation rather than novelty alone. Therefore, it is crucial to design transgene-free pipelines as integrated systems, pairing delivery with built-in enrichment/selection, expanding genotype-robust regeneration platforms, and adopting harmonized molecular verification and multi-generation stability benchmarks. These steps would allow “transgene-free” to become a reproducible outcome for breeding and deployment rather than an isolated proof-of-concept.

In recent years, the rapid advancement of epigenetics has provided a new strategy to achieve traceless genome editing in plants. Unlike RNP delivery, which alters DNA sequences, epigenetic modifications, such as DNA methylation, histone modification, and acetylation, do not alter DNA sequences, making them a viable option for traceless, transgene-free editing. This technique has been successfully employed for the targeted editing of various promoters in *Arabidopsis* using the dCas9-SunTag system, providing a promising method for modulating gene expression and enhancing environmental adaptability [86]. However, the stability and heritability of such epigenetic modifications remain variable and context-dependent, with many modifications gradually reverting in subsequent generations in the absence of sustained targeting. Although epigenetic modification has the potential to become an important tool for genome editing in plants, this system still requires further refinement, as the epigenetic modification footprint caused by the dCas9-SunTag system, along with its off-target effects, pose major challenges [[86], [140], [141], [142]] (Table 2).

Future advances will likely arise from the continued development and integration of several components: data-driven design (including assistance from artificial intelligence [AI]), innovative tools for delivery (including biodegradable nanocarriers), and improvements in plant regeneration (including methods that reduce reliance on tissue culture). At the same time, engineering constraints, such as the cytotoxicity of nanomaterials, the high cost for their synthesis, and difficulty in their controllable release, are major considerations [[81], [143], [144]]. Within a data-driven design framework, AI should help researchers optimize the pipeline from editing components to experimental parameters to screening workflows in a predictable manner. Deep learning models can support the potential off-target risks and the efficiency of base editing, thereby reducing trial-and-error during sgRNA selection and the setting of editing windows [145]. For prime editing, predictive models that learn design rules for pegRNAs, PBS, and RTT are already reducing our dependence on inaccurate, empirical approaches [146]. In parallel, protein language models have been used to guide rational optimization of key prime-editing components, suggesting the potential for improved fidelity and expanded editing windows [147].

Beyond DNA sequence editing approaches, sequence-independent strategies based on epigenetic regulation are increasingly recognized as a complementary direction for transgene-free editing. Tools for editing DNA methylation patterns in plants are becoming more systematic and controllable. For example, researchers have established a combinable, tunable DNA methylation editing platform in *Arabidopsis*, enabling the precise control of methylation states at defined genomic loci through the coordinated design of effector proteins and targeting modules [148]. Rather than focusing on individual phenotypic outcomes, these studies emphasize the development of epigenome editing as a reproducible and generalizable framework, bringing it closer to incorporation into breeding workflows. Nevertheless, epigenetic editing must still be assessed using the same criteria applied to sequence-based editing, including locus specificity, the stability and reversibility of editing effects, and inheritance across generations, before it can be considered a scalable, transgene-free solution [149].

Looking further ahead, “design–delivery–regeneration/screening” data streams could be integrated with multi-omics, phenomics, and genomic-selection platforms [150], making transgene-free editing easier to perform using routine breeding pipelines, thereby lowering validation costs while improving heritable output stability and turning programmable editing into a quantifiable process [151,152]. Such steps could lead to an incremental convergence toward model-driven design–build–test–learn cycles, where precise editing becomes standardized rather than being a replacement for classical breeding. Various components are needed to make these cycles a reality: high recovery rates, molecular cleanliness, stable inheritance, and the realistic treatment of crop-to-crop variability.

Finally, it will be important to design low-barrier transgene-free workflows for use in resource-limited settings. Affordable, cost-effective, quick methods for studying plant stress physiology enable high-quality research to be performed in low-income countries and laboratories with limited resources [153]; the same principle can apply to transgene-free genome editing. Prioritizing delivery and screening options that require less specialized equipment, reduce per-sample molecular burden, and offer scalable readouts (e.g., streamlined genotyping and scalable molecular detection) could help prevent transgene-free editing from being restricted to high-resource locations without relaxing standards for molecular validation. Such accessibility could broaden the availability of data, expand crop and trait coverage, and accelerate locally relevant trait improvement.

## CRediT authorship contribution statement

**Yue-Hao Gao:** Writing – review & editing, Writing – original draft, Visualization, Data curation, Conceptualization. **Qi Lin:** Writing – review & editing, Writing – original draft, Visualization, Data curation, Conceptualization. **Yu-Jing Wang:** Writing – original draft, Data curation, Conceptualization. **Yu Cao:** Writing – review & editing, Writing – original draft, Investigation, Data curation, Conceptualization. **Xin-Xin Li:** Writing – original draft, Visualization, Software, Conceptualization. **Dian-Chen Yue:** Writing – original draft, Resources, Data curation, Conceptualization. **Hou-Ling Wang:** Writing – review & editing, Writing – original draft, Supervision, Resources, Project administration, Investigation, Funding acquisition, Data curation, Conceptualization.

## Declaration of competing interest

The authors declare that they have no conflict of interest.

## Data Availability

No data was used for the research described in the article.
